# Transmission dynamics and epidemiological characteristics of SARS-CoV-2 Delta variant infections in Guangdong, China, May to June 2021

**DOI:** 10.2807/1560-7917.ES.2022.27.10.2100815

**Published:** 2022-03-10

**Authors:** Min Kang, Hualei Xin, Jun Yuan, Sheikh Taslim Ali, Zimian Liang, Jiayi Zhang, Ting Hu, Eric HY Lau, Yingtao Zhang, Meng Zhang, Benjamin J Cowling, Yan Li, Peng Wu

**Affiliations:** 1 Guangdong Provincial Center for Disease Control and Prevention, Guangzhou, Guangdong, China; 2 WHO Collaborating Centre for Infectious Disease Epidemiology and Control, School of Public Health, Li Ka Shing Faculty of Medicine, The University of Hong Kong, Hong Kong Special Administrative Region, China; 3 Guangzhou Center for Disease Control and Prevention, Guangzhou, Guangdong, China; 4 Laboratory of Data Discovery for Health Limited, Hong Kong Science Park, New Territories, Hong Kong Special Administrative Region, China; 5 Foshan Center for Disease Control and Prevention, Foshan, Guangdong, China

**Keywords:** China, emerging or re-emerging diseases

## Abstract

**Background:**

The Delta variant of SARS-CoV-2 had become predominant globally by November 2021.

**Aim:**

We evaluated transmission dynamics and epidemiological characteristics of the Delta variant in an outbreak in southern China.

**Methods:**

Data on confirmed COVID-19 cases and their close contacts were retrospectively collected from the outbreak that occurred in Guangdong, China in May and June 2021. Key epidemiological parameters, temporal trend of viral loads and secondary attack rates were estimated. We also evaluated the association of vaccination with viral load and transmission.

**Results:**

We identified 167 patients infected with the Delta variant in the Guangdong outbreak. Mean estimates of latent and incubation period were 3.9 days and 5.8 days, respectively. Relatively higher viral load was observed in infections with Delta than in infections with wild-type SARS-CoV-2. Secondary attack rate among close contacts of cases with Delta was 1.4%, and 73.1% (95% credible interval (CrI): 32.9–91.4) of the transmissions occurred before onset. Index cases without vaccination (adjusted odds ratio (aOR): 2.84; 95% CI: 1.19–8.45) or with an incomplete vaccination series (aOR: 6.02; 95% CI: 2.45–18.16) were more likely to transmit infection to their contacts than those who had received the complete primary vaccination series.

**Discussion:**

Patients infected with the Delta variant had more rapid symptom onset compared with the wild type. The time-varying serial interval should be accounted for in estimation of reproduction numbers. The higher viral load and higher risk of pre-symptomatic transmission indicated the challenges in control of infections with the Delta variant.

## Introduction

The Delta variant (Phylogenetic Assignment of Named Global Outbreak Lineages (Pangolin) designation lineage B.1.617.2) of severe acute respiratory syndrome coronavirus 2 (SARS-CoV-2) is a variant first detected in India on 7 September 2020 [1]. It was classified by the World Health Organization as a variant of concern (VOC) on 11 May 2021 and had rapidly outcompeted other variants of SARS-CoV-2 by November 2021, with more than 98% of new infections globally caused by the Delta variant in November 2021 [2].

Compared with the wild-type virus, the Delta variant has nine or 10 characteristic mutations, i.e. T19R, G142D, 156del, 157del, R158G, L452R, T478K, D614G, P681R and D950N, which could be responsible for competitive advantages against other variants [3]. The residue 452 spike mutation located at the receptor-binding domain may increase capability of immune evasion and resistance to antibody neutralisation, and P681R in the S1/S2 regions of the S gene could influence proteolytic processing [4]. All these mutations could result in increased affinity for the ACE2 receptor and resistance to antibody neutralisation therefore leading to increases in transmissibility [4].

On 21 May 2021, the first local infection with the Delta variant in mainland China was identified in Guangdong province. A local outbreak occurred in the following days and weeks, and the gene sequence analysis showed that all cases identified in this outbreak were infected with the Delta variant and could be traced back to the index case [5]. An aggressive case finding strategy including multiple comprehensive large-scale nucleic acid tests in high-risk settings (once a local outbreak is identified, locations were classified as low, moderate and high risk for transmission), routine PCR testing for close contacts quarantined in designated places and nucleic acid screening among inpatients and outpatients in areas with local infections detected had been strictly implemented, aiming to identify all infected persons and rapidly control this outbreak. This provides rich epidemiological data on infections with the Delta variant. In this retrospective study, we aimed to explore the transmission dynamics and epidemiological characteristics of the Delta variant outbreak in Guangdong. As the coverage of coronavirus disease (COVID-19) vaccination has increased substantially in China since March 2021, we were able to examine the associations between vaccination and virus shedding and transmission.

## Methods

### Data collection

We retrospectively collected information on all laboratory-confirmed symptomatic and asymptomatic COVID-19 cases with Delta variant infection from the outbreak in Guangdong province in May and June 2021. COVID-19 cases were individuals with a positive result in a PCR for SARS-CoV-2 in respiratory specimens. To estimate the latent period distribution, i.e. the time interval between infection and becoming infectious, we collected individual information on the first (lower bound of possible infection time) and last (upper bound of possible infection time) dates of exposure (infection window), and laboratory testing dates of the last negative PCR test (lower bound of possible start of viral shedding) and the first positive PCR test (upper bound of possible start of viral shedding) which provided a time window when detectable virus shedding began. We also obtained illness onset dates and infection window (same as the one used in latent period estimation) for estimation of the incubation period which describes the time delay from infection to symptom onset. 

Using the information from contact tracing, we reconstructed the transmission pairs from available illness onset dates for both infectors and infectees to estimate the infectiousness profile (daily probability of transmission during the infectious period), the proportion of transmission occurring before symptom onset and the serial interval distribution (time interval of symptom onsets between two successive cases in a transmission chain). Severity status, i.e. asymptomatic, mild, moderate, severe and critical, was collected for each case, along with other information such as sex, age, pre-existing underlying conditions, vaccination status and exposure duration. Cases who had completed the primary vaccination series were defined as individuals who had received two doses of the inactivated vaccines (BIBP COVID-19, Sinopharm, Beijing, China or CoronaVac, Sinovac Biotech Ltd, Beijing, China) 14 days or longer before the first day of possible exposure to an infector. Cases who had received an incomplete vaccination series were defined as having had one vaccination dose 14 days or longer before the first day of possible exposure to an infector. We also collected information on close contacts of cases infected with the Delta variant to estimate secondary attack rates and identify predictors of infection.

Comprehensive large-scale PCR testing strategies, including compulsory testing in high-risk settings, routine testing among close contacts quarantined in designated places (often quarantine hotels) and daily testing for inpatients, were implemented in every local COVID-19 outbreak in China since April 2020 [6]. For each case, serial samples were collected and tested for both ORF1ab and N genes from the date of first positive PCR test until discharge from hospital. Different brands of PCR test kits targeting the same genes (ORF1ab and N) with the same diagnostic quantification cycle (Cq) threshold were used for the diagnosis of infection with SARS-CoV-2 (Supplementary Table S1 provides details on the test kits used in this study). To understand the temporal dynamics of viral RNA shedding for the Delta variant, we obtained serial Cq threshold values for the N gene for each case with throat swabs from the first positive test (Cq < 40). To compare viral loads between the Delta variant and the wild type, we used individual data with Cq values from serial PCR tests conducted during hospital isolation on infected patients with the SARS-CoV-2 ancestral virus (which we refer to as wild type) identified in Hong Kong in early 2020. Additional information about detailed case definitions used in this study is provided in the Supplement.

### Statistical analysis

We used a Bayesian inferential method to estimate the distributions of latent period, incubation period, serial interval and the infectiousness profile of confirmed COVID-19 cases by fitting Weibull, gamma and lognormal distributions, and the distribution with lowest leave-one-out information criterion (LOO IC) was selected in the final simulation. The latent period, incubation period and infectiousness profile distributions were jointly estimated because they used the same exposure window. Non-negative flat prior probability distributions for the parameter values of the three distributions were specified. We accounted for the interval censoring of exposure and viral shedding windows when estimating the latent period and incubation period distributions and infectiousness profile. We used a uniform prior probability distribution over the exposure interval and viral shedding windows for the time of infection and start of virus shedding for each case. We sampled from the posterior distributions using the *rstan* package in R software (R Foundation for Statistical Computing, Vienna, Austria).

We estimated the time-varying forward serial intervals by using the method provided by Park et al. [7]. The time-varying forward serial intervals [7,8] and daily numbers of cases were used to estimate the daily instantaneous reproduction number (R_t)_ by applying the statistical methods used in the *EpiEstim* package developed by Cori et al. [9]. The daily incidence data during the exponential growth phase was used to obtain the exponential growth rate using the *incidence* package in R software. The initial forward serial interval distribution was obtained by using mean estimates of the serial intervals during the exponential growth phase of the Delta outbreak [7]. The basic reproduction numbers (R_0_) were estimated based on the forward serial intervals and exponential growth rate during the exponential growth phase using the method suggested by Park et al. [7]. Details and equations are provided in the Supplement.

The overall temporal trend of Cq values for the N gene in COVID-19 cases infected with the Delta variant was analysed by day of illness onset. To aid visualisation, smoothing splines using a generalised additive model (GAM), including days of illness onset as the only predictor, were fitted to the Cq values to characterise the overall trend for the Delta variant. To make a comparison between Delta and wild type, we also fitted the temporal trend of Cq values for the Delta variant and wild type separately. To evaluate the impact of COVID-19 vaccination on viral loads among cases infected with the Delta variant, we fitted a multivariate GAM by including variables of vaccination (1: without vaccination, 2: with complete or incomplete vaccination series), days of illness onset, age and disease severity. Temporal trend of predicted Cq values from the GAM was presented and compared using box plots for vaccinated and unvaccinated cases separately.

Close contacts of confirmed COVID-19 cases infected with the Delta variant with a sole possible source of infection for each close contact were included for analysis. The overall secondary attack rate was calculated by dividing the number of infections by the total number of close contacts. To assess the effectiveness of vaccination against transmission, a stepwise logistic regression model was fitted by including age, sex, disease severity of the index, COVID-19 vaccination for index cases, COVID-19 vaccination for close contacts, type of contact, presence of exposure on the symptom onsets of index cases and duration of exposure. A non-parametric and parametric bootstrap approach with 1,000 resamples was used to assess the uncertainty of each estimated parameter. Analyses were carried out using R version 4.1.0. All anonymised data collected are publicly available at https://github.com/XinHKU/Delta-variant/tree/main.

### Ethical statement

This study was approved by the institutional ethics committee of the Guangdong Provincial Center for Disease Control and Prevention (GDCDC) on 6 July 2021. Written informed consent was not collected from the patients since the data used in this study were anonymised.

## Results

By 18 June 2021, 167 SARS-CoV-2 Delta infections had been identified in the outbreak in Guangdong. Sixty-nine (41.3%) were male. The median age was 47.0 years (interquartile range (IQR): 31.0–66.5) with 22 (13.2%) cases younger than 15 years and 44 (26.3%) older than 64 years. The number of asymptomatic, mild, moderate and severe or critical cases was eight (4.8%), 29 (17.4%), 111 (66.5%) and 19 (11.4%), respectively, with no reported deaths. Sixteen (9.6%) cases had received the complete primary vaccination series and 30 cases (18.0%) had received an incomplete vaccination series.

**Figure 1:**
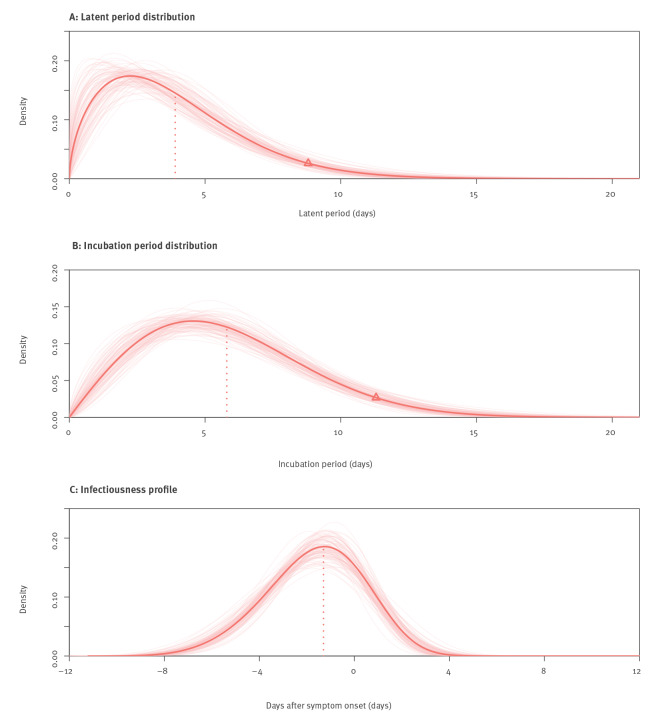
Key epidemiological time-delay distributions of SARS-CoV-2 Delta variant, Guangdong, China, May–June 2021 (n = 93 transmission pairs)

We examined data from 93 Delta transmission pairs with sufficient information to estimate the latent period, incubation period, infectiousness profile and serial interval, and the Weibull distribution provided the best fit for the data (see Supplementary Table S2 for comparison of different distributions). The mean latent period was estimated to be 3.9 days (95% credible interval (CrI): 3.3–4.5). Ninety-five per cent of the cases with Delta started shedding virus within 8.8 days (95% CrI: 7.5–10.7) after infection (Figure 1A). The mean incubation period estimated from 93 symptomatic cases with Delta was 5.8 days (95% CrI: 5.1–6.5). The 95th percentile of the incubation period for Delta was 11.3 days (95% CrI: 10.2–12.9) (Figure 1B). We used the same data set to estimate the infectiousness profile allowing for transmission before symptom onset. We estimated that 0.7% (95% CrI: 0.5–3.3) of transmissions occurred more than 7 days before illness onset, 11.9% (95% CrI: 2.1–28.3) cases started to become infectious 4 days before illness onset, and the infectiousness peaked at 1.3 days (95% CrI: 0.8–1.8) before onset and then dropped gradually, with 73.1% (95% CrI: 32.9–91.4) of the transmissions occurring before illness onset and 99.8% (95% CrI: 93.2–100.0) of transmissions occurring within 4 days after illness onset (Figure 1C).

SARS-CoV-2: severe acute respiratory syndrome coronavirus 2.

Red lines: distributions using mean parameters (shape and scale). We sampled 100 parameters for each distribution to indicate uncertainty (pink lines). Vertical dotted lines: mean estimates for latent period distribution and incubation period distribution, and peak estimate for infectiousness. Triangles in panels A and B: 95th percentiles for respective parameters.

**Figure 2:**
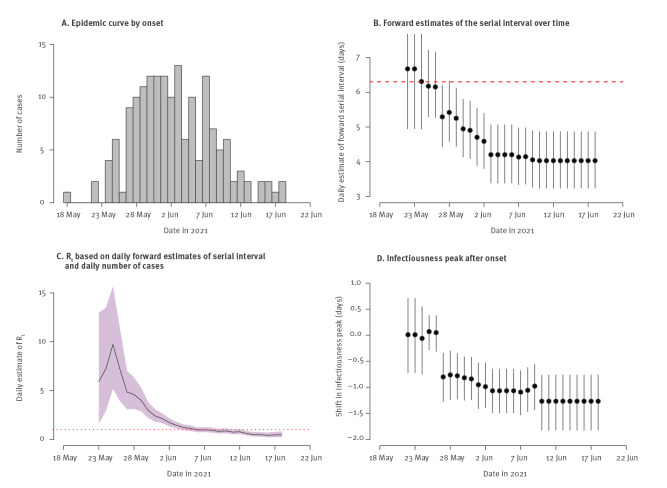
The estimated daily forward serial intervals and instantaneous reproduction numbers and infectiousness peak during the SARS-CoV-2 Delta outbreak in Guangdong, China, May–June 2021 (n = 93 transmission pairs)

CrI: credible interval; R_t_: instantaneous reproduction number; SARS-CoV-2: severe acute respiratory syndrome coronavirus 2.

The estimated forward serial intervals decreased from 6.7 days (95% CrI: 4.9–9.3) on 22 May 2021 to 4.0 days (95% CrI: 3.2–4.9) on 18 June 2021 in the Delta outbreak (Figure 2B). By using the time-varying forward serial intervals and case incidence data, we estimated that the R_t_ increased from 5.9 (95% CrI: 1.6–13.0) on 23 May 2021 to 9.7 (95% CrI: 5.2–15.7) on 25 May 2021, then dropped rapidly to 0.49 (95% CrI: 0.23–0.83) on 18 June 2021 and had been below 1 since 6 June 2021 (Figure 2C). During the same time period, the estimated infectiousness peak shifted from 0.01 days (95% CrI: −0.72 to 0.71) after illness onset to 1.26 days (95% CI: 0.76–1.82) before illness onset (Figure 2D). By using the daily estimates of forward serial interval and exponential growth rate (0.33; 95% CrI: 0.18–0.48) (see Supplementary Figure S1 for a detailed representation of exponential growth rate) during the exponential growth phase before 26 May 2021, the initial forward serial interval was estimated to be 6.3 days (95% CrI: 5.1–8.4) (Figure 2B), and the R_0_ was 4.9 (95% CrI: 3.1–6.5).

Panel A is based on symptom onset dates of all cases in the Delta outbreak in Guangdong province. Panel B: dots represent the daily mean estimates of serial interval and the vertical line segments represent the 95% CrI; the dashed line represents the initial (from transmission pairs with illness onsets of infectors before 26 May 2021) forward serial interval (6.3 days). Panel C: the line represents mean estimates of R_t_ with 95% CrI (shaded area) and the dotted line indicates R_t_ = 1. Panel D: dots represent the daily mean estimates of time of infectiousness peak in comparison with the onset day with the 95% CrI (vertical line segments).

**Figure 3:**
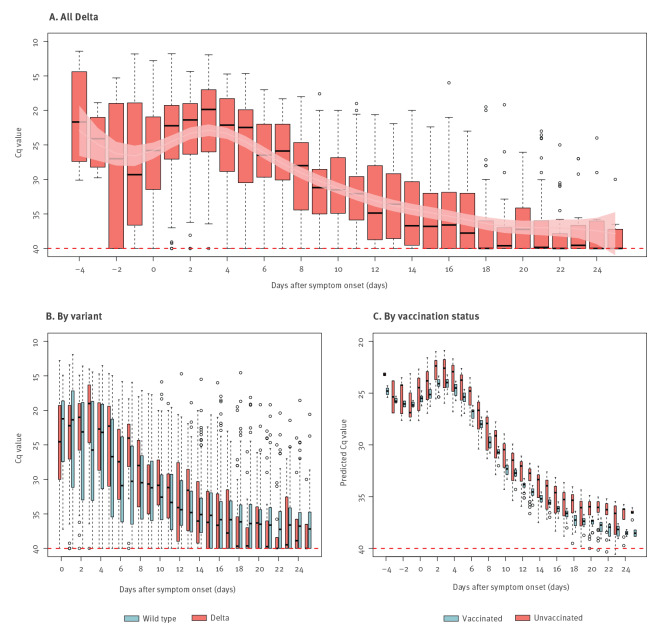
Temporal patterns of viral shedding in cases infected with the SARS-CoV-2 Delta variant in Guangdong, May–June 2021 (n = 159) and the wild type in Hong Kong, February–April 2020 (n = 751)

In total, 1,312 throat swabs collected between 4 days before and 34 days after illness onset were tested for 159 cases with SARS-CoV-2 Delta infection in Guangdong. High viral loads were maintained between 4 days before onset and 7 days after onset, then decreased gradually to a low but detectable level until about Day 20 (Figure 3A). To compare viral loads between Delta and wild type, we identified 751 cases infected with the wild-type virus in Hong Kong between 12 February 2020 and 16 April 2020. None of these cases had received COVID-19 vaccination. In total 8,008 respiratory samples were collected and tested on the illness onset day and 31 days after onset for the 751 wild-type cases. We found during the period with a high viral load (0 to 7 days after onset), the median Cq values were 23.0 (IQR: 19.3–28.6) for the N gene of the Delta variant, significantly lower than the values of the wild-type N gene (median: 26.9; IQR: 20.9–34.3) (Figure 3B). Results of the GAM revealed that the Cq values of the 46 Delta cases with complete and incomplete vaccination series were on average 0.97 (95% CI: 0.19–1.76) higher than unvaccinated cases after adjusting for days of illness onset, age and disease severity (Figure 3C).

Cq: quantification cycle; GAM: generalised additive model; SARS-CoV-2: severe acute respiratory syndrome coronavirus 2.

Panel A: Boxplot of Cq for the N gene for all cases infected with the Delta variant. Light red curve and light pink shaded area indicate the fitted Cq values and the 95% confidence intervals estimated from the GAM. Red dashed line: limit of detection (Cq = 40).

Panel B: Boxplot of Cq values for the N gene of Delta and wild type against time of sample collection relative to the date of illness onset.

Panel C: Boxplot of the predicted Cq values from the multivariate GAM for unvaccinated and vaccinated (with one or two doses) cases infected with the Delta variant.

To evaluate individual infection risk and the effectiveness of vaccination against transmission of the Delta variant, we analysed infections in 5,153 individuals who were close contacts of 73 COVID-19 cases. The overall secondary attack rate was 1.4% (95% CI: 1.1–1.8) in the contacts and the rate was highest among household and extended family (22.0%; 95% CI: 16.2–28.3). The stepwise regression model showed that the infection risk increased with age (adjusted odds ratio (aOR): 1.02; 95% CI: 1.01–1.03), and the infection risk was high for exposure to an index case without vaccination (aOR: 2.84; 95% CI: 1.19–8.45) or with incomplete vaccination (aOR: 6.02; 95% CI: 2.45–18.16) and for household and extended family contacts (aOR: 39.90; 95% CI: 24.15–66.24) (Table).

**Table:** Secondary attack rate for close contacts of the SARS-CoV-2 Delta infectors and risk factors associated with the occurrence of infection based on backward logistic regression, Guangdong, China, May–June 2021 (n = 5,153)

Characteristics of contacts	Close contacts	Infections	SAR (%)	Adjusted OR (95% CI)
Overall	5,153	73	1.4	NA
**Sex**				
Male	2,553	29	1.1	NA
Female	2,600	44	1.7	NA
**Age in years: median (IQR)**	38.00 (27.00–52.00)			1.02 (1.01–1.03)
≥ 0	424	12	2.8	NA
≥ 15	2,683	18	0.7	NA
≥ 45	1,521	27	1.8	NA
≥ 65	525	16	3.0	NA
**Type of index cases**				
Asymptomatic and mild	1,809	12	0.7	NA
Moderate, severe or critical	3,344	61	1.8	NA
**COVID-19 vaccine dose of index cases**				
0	2,892	37	1.3	2.84 (1.19–8.45)
1^a^	1,110	31	2.8	6.02 (2.45–18.16)
2^b^	1,151	5	0.4	Reference
**COVID-19 vaccine dose of contacts**				
0	2,844	48	1.7	NA
1^a^	1,459	17	1.2	NA
2^b^	850	8	0.9	NA
**Type of contact**				
Household and extended family	173	38	22.0	39.90 (24.15–66.24)
Others	4,980	35	0.7	Reference
**Exposure to an index case at onset^c^**				
Yes	2,106	49	2.3	NA
No	3,047	24	0.8	NA
**Duration of exposure in days: mean (SD)**	7.78 (3.83)			
≥ 1	1,092	7	0.6	NA
≥ 6	4,061	66	1.6	NA

CI: confidence interval; IQR: interquartile range; NA: not applicable; OR: odds ratio; SAR: Secondary attack rate; SARS-CoV-2: severe acute respiratory syndrome coronavirus 2; SD: standard deviation.

^a^ First COVID-19 vaccine dose 14 days or longer before the first day of possible exposure to an infector (incomplete vaccination series).

^b^ Second COVID-19 vaccine dose was given 14 days or longer before the first day of possible exposure to an infector (complete primary vaccination series).

^c^ Close contacts were exposed to an index at the time of symptom onset of the index.

## Discussion

Our study provided a comprehensive assessment of the epidemiological characteristics of the SARS-CoV-2 Delta variant during an outbreak. Higher transmissibility was demonstrated for the Delta variant, as indicated by a higher reproduction number and shorter latent and incubation periods compared with the wild-type SARS-CoV-2 [5,8,10-13]. We observed higher viral loads in cases infected with the Delta variant, which might contribute to more rapid and intense transmission. In addition, we found that a complete primary vaccination series of the inactivated vaccines, i.e. Sinopharm BIBP COVID-19 inactivated vaccine or Sinovac-CoronaVac inactivated vaccine could effectively reduce viral loads in cases infected with the Delta variant and further lead to lower transmissibility.

We estimated the time-varying forward serial intervals which considered the temporal dynamics of disease transmission in an outbreak [7,8]. The R_0_ estimated for the Delta variant was 4.9 which was substantially higher than the R_0_ of the ancestral virus (2.2) at the start of the pandemic [10,14]. Studies about the Alpha (B.1.1.7) variant found the ratio of reproduction numbers between Alpha variant and non-VOC to be between 1.54 and 1.89 [15], while our study showed that the ratio between Delta variant and wild-type was 2.2, consistent with a slightly higher R_0_ of Delta variant compared with Alpha variant. Estimation of the reproduction number could be underestimated because of unobserved infections and if the changes in the forward-serial interval distribution during the period of epidemic were neglected [7]. In the Delta outbreak in Guangdong, active and aggressive case-finding strategies using PCR tests were implemented, which enabled identification of most infected persons including asymptomatic cases. With the shorter latent and incubation periods, and higher secondary attack rate among household and extended family contacts, we believe multiple and stringent interventions are needed to control epidemics of the Delta variant. During the Delta outbreak in Guangdong, the local government had implemented individual-based interventions such as case isolation, contact tracing and quarantine, as well as population-level physical distancing measures such as lockdowns and confinement [8,16]. More importantly, various community-wide PCR testing and routine testing programmes among quarantined close contacts were aligned with the measures of contact tracing and lockdown, aiming to identify and isolate the cases as early as possible and interrupt transmission chains. The rapid drop in R_t_ within a week indicated the effectiveness of these interventions.

We estimated that 73.1% of transmissions occurred pre-symptomatically for the Delta variant in this outbreak, which was higher than for other variants [17-19], suggesting a higher transmission potential of Delta before detection; this was further supported by the high viral loads (limit of detection: Cq = 40) at least 4 days before illness onset shown in our study. The high risk of transmission particularly before onset indicated the need to expand contact tracing to a wider group of contacts and perhaps to a longer time scale in order to control an outbreak caused by the Delta variant [18,20]. However, for areas with a high incidence of COVID-19, complete contact tracing and quarantine outside the home may not be feasible as the number of contacts is always several times the number of infections [16]. Physical distancing such as self-isolation and home quarantine is more suitable in these areas. However, society-wide physical distancing measures might increase transmission risk in household settings [16,17]. Our study showed that the secondary attack rate (22.0%) among close household contacts of cases infected with the Delta variant was higher than the secondary attack rate obtained in 2020 (12.4%) in the same location for infections with ancestral strain [21]. It was also higher than the secondary attack rate of wild-type SARS-CoV-2 reported in a systematic review about household transmission (13.4%) [22]. However, the difference was not so obvious in a comparison with the Alpha variant (24.5%) [22].

We found that the viral load was higher in cases with the Delta variant than compared with the wild-type virus, indicating a potentially higher infection rate per contact for Delta [23]. In addition, patients infected with the Delta variant maintained a high viral load from 4 days before illness onset. Escape of the Delta variant from immunity induced by the wild type [24] suggests that the level of population immunity provided by a complete primary vaccination series before the epidemic of Delta variant may not be sufficient to control spread of the Delta variant [23]. Additional booster doses of vaccination may be able to increase protection against the later variants.

The effectiveness of other vaccines appeared to diminish against infections with the Delta variant compared with other variants [24,25]. However, the effectiveness of COVID-19 vaccines against transmission, which is another important indicator of their impact [26], has rarely been reported. In this study, we observed that the Cq values among cases infected with SARS-CoV-2 Delta after vaccination with one or two doses of inactivated vaccines were on average 0.97 higher than among unvaccinated cases, indicating a threefold decrease in viral RNA copies [27]. The vaccinated cases with Delta in our study with a decreased viral load may have had a reduced transmission potential given that the viral RNA load of SARS-CoV-2 was independently associated with virus shedding [28], which was consistent with the analysis of secondary attack rates among close contacts. Interestingly, we found that the index cases with incomplete vaccination series were more likely to infect their close contacts than those without vaccination. This might be attributed to the behavioural changes in people with one dose vaccination who were perhaps more likely to have more relaxed physical distancing behaviours than the unvaccinated individuals. Our study also found that the secondary attack rate among close contacts who completed two-dose vaccination was half that in the unvaccinated, although the difference was not statistically significant. One case–control study which was based on the same outbreak estimated that the overall vaccine (same type of vaccine as in our study) effectiveness was 59.0% (95% CI: 16.0–81.6) [29]. The effectiveness of inactivated vaccines against transmission of the Delta variant demonstrated the importance of increasing vaccination coverage in mitigating COVID-19 [30].

Our study had several limitations. Firstly, self-reported symptom onset might bias estimates of the parameters, e.g. leading to an overestimation of the incubation period if patients tended to remember the later days with symptoms. Uncertainty of the time of infection and detectable RNA shedding and onset of contagiousness would have decreased the precision of the estimated parameters. Secondly, the patients included in our study may have been diagnosed with PCR test kits from different brands, which might lead to measurement differences in the Cq values analysed in our study. However, we believed that this would not affect our findings substantially given the fact that these different test kits targeted the same genes (ORF1ab and N) with the same diagnostic threshold, and that the Cq values for the same patients from the two most frequently used PCR test kits were generally agreeable with each other (see Supplementary Table S3 for Cq values from different PCR test kits). Thirdly, in estimation of the serial interval, transmission pairs with asymptomatic cases would be excluded if the date of symptom onset was unavailable, which might have biased the estimates of the reproduction number by neglecting the impact of asymptomatic transmission. Finally, the number of transmission pairs used in our study was small which might affect the accuracy of the estimated parameters.

## Conclusion

The Delta variant demonstrated a higher transmissibility compared with the wild type of SARS-CoV-2. An extension of the contact tracing period to perhaps 4 days before symptom onset may be needed considering the high proportion of pre-symptomatic transmission and the high viral load before onset in infections with the Delta variant. Inactivated vaccines appeared to be effective in reducing transmission of Delta infections and a high vaccination coverage should be pursued to reduce the burden of the COVID-19 pandemic.

## Supplementary Data

334000 Supplement
