# Supplementary material for: Transmission dynamics and epidemiological characteristics of SARS-CoV-2 Delta variant infections in Guangdong, China, May to June 2021
Source: Euro Surveill. 2022 Mar 10;27(10):2100815. doi: 10.2807/1560-7917.ES.2022.27.10.2100815 (PMC8915401; doi:10.2807/1560-7917.ES.2022.27.10.2100815)
Supplement: Supplement [file 21-00815_COWLING_Supplement.pdf]

This supplementary material is hosted by Eurosurveillance as supporting information alongside the article [Transmission dynamics and epidemiological characteristics of SARS-CoV-2 Delta variant infections in China], on behalf of the authors, who remain responsible for the accuracy and appropriateness of the content. The same standards for ethics, copyright, attributions and permissions as for the article apply. Supplements are not edited by Eurosurveillance and the journal is not responsible for the maintenance of any links or email addresses provided therein."

Supplementary Materials

Case definitions and detailed methods for parameters estimation

Case definitions

A patient is confirmed as a COVID-19 case based on a positive result of PCR for SARS-CoV-2 with respiratory specimens. Virus strains in this study were determined by the sequenced genome and were classified based on the "Pango lineages" rule (1). The time interval between infection and becoming infectious is defined as the latent period, that could be compared with the incubation period which describes the time duration between infection and symptom onset. The latent period is typically proxied by the time from infection until an infected person has virus shedding that is detectable by PCR, and can be shorter than the incubation period for some COVID-19 cases when virus shedding becomes detectable prior to symptom onset. The serial interval, defined as the time interval between successive symptom onsets in a transmission chain, is an important parameter for estimating many other key epidemiological parameters, such as R_0, the expected number of secondary cases generated from one primary case in a completely susceptible population, and the instantaneous reproduction number (R_t) which describes the expected number of secondary cases caused by one typical primary case at time *t*. The infectiousness profile of COVID-19 describes the duration and intensity of infectiousness of infected cases which imply the probability of transmission during the infectious period. In this study, the distribution of infectiousness profile was estimated as the time interval between the moment of infection for an infectee (case) and the illness onset time of infector (infection source of the case).

We assessed the clinical severity of COVID-19 cases via clinical classification into

asymptomatic, mild, moderate, severe and critical following the Guidelines in Diagnosis and Treatment of COVID-19 (8th version) published by National Health Commission since 15 April 2021 (2). Asymptomatic SARS-CoV-2 infections were patients who tested positive for SARS-CoV-2 without presenting any symptoms potentially related to COVID-19, such as fever, chill, dry cough, nasal congestion, loss of taste or smell, runny nose sore throat, headache, tiredness, muscle pain, joint pain, short of breath, difficulty breathing, conjunctivitis, nausea, vomit, diarrhoea, abdominal pain, etc., and without lung infections indicated by a chest X-ray examination, throughout the course of infection. Mild type is with mild clinical symptoms and without lung infections indicated by a chest X-ray examination. Moderate type means with fever, respiratory track symptoms and with lung infections indicated by a chest X-ray examination. A person was defined as severe if the patient showed one of the criteria: (1) shortness of breath; (2) Oxygen saturation ≤93% when inhaling air at rest condition; (3) PaO_2/FiO_2 <=300mmHg (1mmHg=0.133kPa); (4) The clinical symptoms were progressively aggravated, and the lung imaging showed significant lesion progression >50% within 24 ~ 48 hours. A person was defined as critical if the patient showed one of the criteria: (1) Respiratory failure and mechanical ventilation is required; (2) Shock; (3) Combined other organ failure and ICU care is required;

Close contacts were defined as individuals who were exposed to symptomatic COVID-19 cases within two days before their illness onset, or exposed to asymptomatic cases at close proximity (<1 meter) without wearing proper personal protection equipment

within two days before their sampling dates of the first positive samples for SARS-CoV-2. Close contacts were classified as household and extended family, social, community and healthcare contacts based on the definitions previous published by *Sun et al* (3). Cases were considered having effective 1-dose vaccination if the start date of exposure was 14 days after the first dose of vaccination or later, or having effective 2-dose vaccination if the start date of exposure was 14 days after the second dose of vaccination or later (4, 5).

Estimation of time-varying parameters

1. Estimating time-varying forward serial intervals

For the 93 transmission pairs we constructed, we rearrange the line list based on the symptom onset dates of infectors. The forward serial interval distribution was estimated by using a cohort of infectors who shared the same symptoms onset date. Then, we estimated the time-varying forward serial interval as we incorporate more recent cohorts into the analysis. That is, we estimated the forward serial interval from infectors who presented symptoms before time *t* and evaluate how the estimates of time-varying forward serial intervals change by increasing *t*. The initial serial interval was estimated based on cohorts of infectors who shared the same symptom onset dates in the exponential phase of outbreak. The initial forward serial interval was obtained before implementing control measures thus would reflect the real serial interval of the Delta variant and therefore can be used to estimate the unbiased R_0 (6, 7).

2. Estimating daily R_t

By using the time-varying forward serial intervals and daily incidence number, we estimated Rt by using the following equation suggested by *Cori et al (8).*

$$R_t = \frac{I_t}{\sum_{i=1}^{t} I_{t-i} w_i}$$

Where I_t is the number of cases in time t, w_i is the serial interval distribution that approximates the infectiousness profile of an infected individual at i-th day since infection. Then, the Rt was calculated as the ratio between the number of cases I_t on day t and the weighted average of infectiousness caused by cases infected before day

t i.e., $\sum_{i=1}^{t} I_{t-i} w_i$.

3. Estimating R_0

We estimated R_0 by using the following equation provided by *Park et al (6)*.

$$\frac{1}{R_0} = \int_{-\infty}^{\infty} exp\ (-r\tau) f_0(\tau) d\tau$$

Where r was the exponential growth rate which we obtained by using the daily number of cases reported in the Guangdong COVID-19 outbreak (**Figure S1**), τ was the time-varying forward serial intervals during the exponential growth phase and $f_0(\tau)$ was the forward serial interval distribution.

Table S1. The numbers of patients tested by different type of test kit for diagnosis of SARA-CoV-2 infection.

Type of kit	No. of patients	%. of all patients
“Daan”	58	36.5
“Shuoshi”	51	32.1
“Mingde”	27	17.0
“Bojie”	11	6.9
Unclassified	81	50.9

Table S2. Alternative parametric estimates of the latent period and incubation period.

Distribution	Incubation period		Latent period		Infectiousness profile		Loo IC[b]
	Mean[a]	SD	Mean	SD	Mean	SD	
Lognormal	5.8	3.3	4.0	2.8	-1.5	3.6	1384.0
Gamma	5.7	2.9	3.9	2.4	-1.7	2.8	1364.5
Weibull	5.8	3.0	3.9	2.6	-1.7	2.1	1343.7

[a] Mean: Posterior mean

[b] Loo IC: Leave-one-out information criterion, Indicates the goodness-of-fit, a lower value indicates a better model fit.

Table S3. Wilcoxon rank sum test for Cq values (N gene) among the 32 patients tested by both “Daan” and “Shuoshi”.

Type of kit	Cq value (Median, IQR)	*P* value
“Daan”	30.1 (23.5-33.4)	0.42
“Shuoshi”	28.8 (21.8-32.7)	

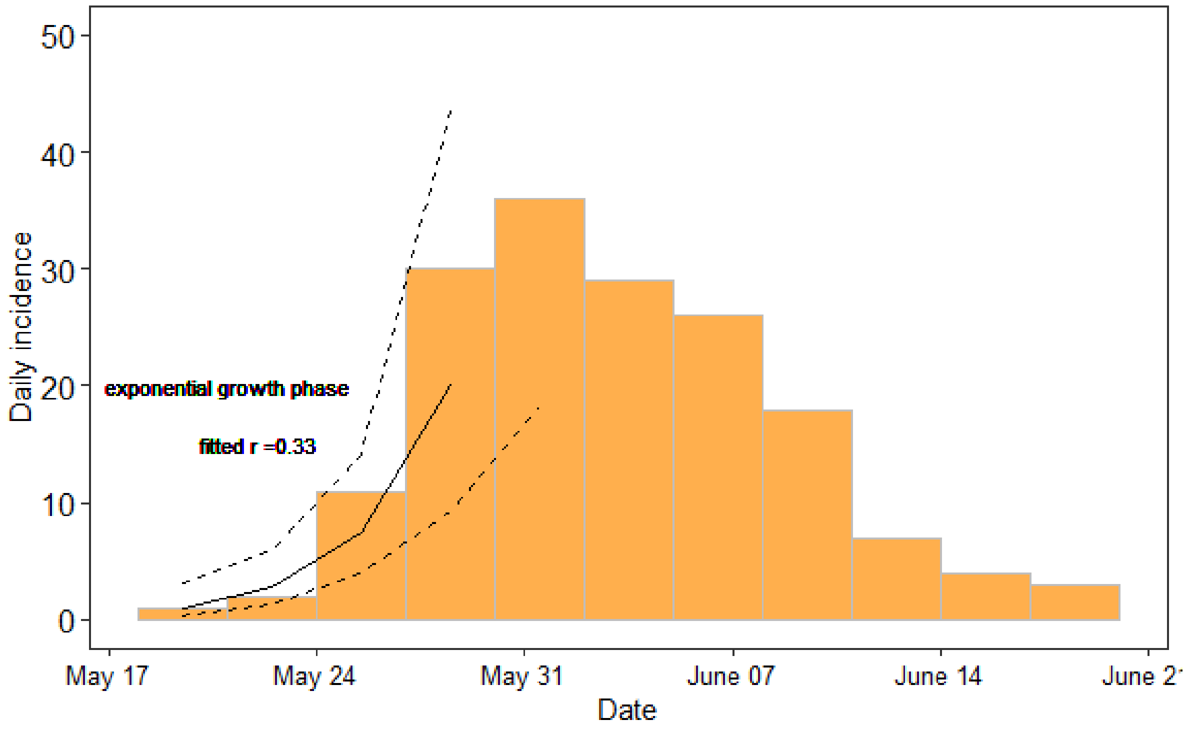

Figure S1. Epidemic curve of the Guangdong COVID-19 cases during May 18, 2021 and June 18, 2021, with exponential growth curve and their 95% confidence interval. The daily incidence is quite noisy for exponential growth rate estimation, so we did the estimation by using incidence with 3 days intervals.

References

1. Rambaut A, Holmes EC, O'Toole Á, Hill V, McCrone JT, Ruis C, et al. A dynamic nomenclature proposal for SARS-CoV-2 lineages to assist genomic epidemiology. Nat Microbiol. 2020;5(11):1403-7.

2. National Health Commission of the People's Republic of China. Guidelines in Diagnosis and Treatment of COVID-19 (eighth version). (available at http://www.nhc.gov.cn/cms-search/downFiles/a449a3e2e2c94d9a856d5faea2ff0f94.pdf). Accessed 10 July 2021.

3. Sun K, Wang W, Gao L, Wang Y, Luo K, Ren L, et al. Transmission heterogeneities, kinetics, and controllability of SARS-CoV-2. Science. 2021;371(6526).

4. World Health Organization .The Sinopharm COVID-19 vaccine: What you need to know. WHO Strategic Advisory Group of Experts, (available at https://www.who.int/news-room/feature-stories/detail/the-sinopharm-covid-19-vaccine-what-you-need-to-know). Accessed 10 July 2021.

5. World Health Organization .The Sinovac COVID-19 vaccine: What you need to know. WHO Strategic Advisory Group of Experts, (available at https://www.who.int/news-room/feature-stories/detail/the-sinovac-covid-19-vaccine-what-you-need-to-know). Accessed 10 July 2021.

6. Park SW, Sun K, Champredon D, Li M, Bolker BM, Earn DJD, et al. Forward-looking serial intervals correctly link epidemic growth to reproduction numbers. Proc Natl Acad Sci U S A. 2021;118(2).

7. Ali ST, Wang L, Lau EHY, Xu XK, Du Z, Wu Y, et al. Serial interval of SARS-CoV-2 was shortened over time by nonpharmaceutical interventions. Science. 2020;369(6507):1106-9.

8. Cori A, Ferguson NM, Fraser C, Cauchemez S. A new framework and software to estimate time-varying reproduction numbers during epidemics. Am J Epidemiol. 2013;178(9):1505-12.
